# Accuracy of Leg Length and Offset Measurements During Total Hip Arthroplasty Using an Imageless Navigation System

**DOI:** 10.7759/cureus.38689

**Published:** 2023-05-07

**Authors:** Anton P Lambers, Melanie A Marley, Robert Jennings, Andrew Bucknill

**Affiliations:** 1 Orthopaedics, Royal Melbourne Hospital, Melbourne, AUS

**Keywords:** total hip arthroplasty (tha), robotic surgical procedures, orthopaedic navigation, computer navigation, hips

## Abstract

Introduction

Leg length and offset are important considerations in total hip arthroplasty (THA). Navigation systems are capable of providing intra-operative measurements of leg length and offset, and high accuracy has been shown in experimental studies. This study assesses the accuracy of an imageless navigation system with a pinless femoral array (Hip 5.1, BrainLAB, Feldkirchen, Germany) in measuring leg length and offset changes in vivo.

Methods

A prospective, consecutive series of 37 patients undergoing navigated THA were included in the study. Intra-operative measurements of leg length and offset were recorded using the navigation system. For each patient, pre- and post-operative digital radiographs were scaled and analyzed to provide radiographic measurements for comparison.

Results

Measurements of leg length change made by the navigation system showed a strong correlation with the size of change measured radiographically (R = 0.71; p<0.0001). The mean difference between the radiographic and navigational measurement was 2.6mm ± 3.0mm (0.0-16.0mm) (mean, SD, range). The navigation system was accurate to within 1mm of the radiographic measurement in 49% of cases, within 2mm in 66% of cases, and within 5mm in 89% of cases.

Measurements of offset change by the navigation system also showed a correlation with radiographic measurements, albeit less pronounced (R = 0.35; p=0.035). The mean difference between navigational and radiographic measurements was 5.5mm ± 4.7mm (0.0-16.0mm) (mean, SD, range). The navigation system was accurate within 1mm of the radiographic measurement in 22% of cases, within 2mm in 35% of cases, and within 5mm in 57% of cases.

Conclusions

This research demonstrates in vivo that an imageless, non-invasive navigation system is a reliable tool for intra-operative leg length (accurate within 2mm) and to a lesser extent offset measurement (accurate within 5mm) when compared to standard practice of plain film radiographs.

## Introduction

Computer-aided surgery is an expanding field covering many surgical specialties [1]. Research in the field is ongoing [2]; however, the widespread acceptance of navigation is hampered by increased costs, increased time, and a lack of evidence that demonstrates long-term benefits. Over the last few decades, navigated orthopedic operations have expanded considerably [3,4]. The application of navigation to total hip arthroplasty (THA) now allows information on prosthetic range of motion, femoral stem and acetabular cup positioning, changes to leg length and offset, and navigation of hip resurfacing procedures [5,6]. In addition, the surgeon is provided with intra-operative feedback of trial components. Previously, a placement error would not be noticed until post-operative radiographs, on occasion necessitating another operation if secondary complications arise. While robotic systems are increasingly in use to also increase the accuracy of leg length and offset changes, these are more costly.

Leg length is important to consider during THA. Post-operative leg length discrepancy (LLD) can result in lower back pain, sciatic nerve palsy, instability, gait abnormalities, stress fractures, limp, patient dissatisfaction, and litigation [7-10]. Only one study has published results contradictory to this, showing that post-operative LLD had no correlation with post-operative outcome or patient satisfaction [11]. It is difficult to identify when an LLD becomes significant, and patient awareness has a major influence. While the size of the leg length inequality correlates with awareness [12,13], varying perceptions of discrepancy have been described by patients [7,9,11-13]. Up to 10mm is the generally accepted size of LLD [14].

To address LLD, pre-operative assessment is vital, including the patient’s perception of discrepancy and if this is apparent or true [15]. A reliable method of radiographically or clinically evaluating leg length is then employed so that the LLD can be addressed [16]. Patient dissatisfaction with discrepancy is the most common cause of orthopedic litigation [9,12] and accurately reconstructing leg length would not only reduce litigation but also improve patient satisfaction and reduce leg length-related post-operative complaints. Navigation of leg length change aims to improve the surgeon’s ability to accurately restore leg length; however, this is based on the assumption that intra-operative measurements are accurate.

Femoral offset has been shown to influence polyethylene wear (prosthetic longevity), hip stability, soft tissue tensioning, abductor muscle function, joint reaction forces, prosthetic micromotion, prosthetic and interface stresses, and range of motion [17-20]. A lack of offset restoration has also been correlated with an increased risk of post-operative limp and the need for walking aids [21]. The majority of evidence supports the restoration of native femoral offset [17-20].

Intra-operative measurements of leg length and offset made by navigation systems in both experimental and patient studies show varying reliability [6,22-25]. A comparative study assessed the same navigation software in cadavers [6,24]. Comparing measurements made using navigation and computed tomography (CT) scans, Renkawitz et al. demonstrated a mean difference of 0.50mm (95% CI: -0.37 to 1.37mm) for leg length measurements and 0.49mm (95% CI: -0.2 to 1.17mm) for offset measurements [6,24]. This study showed a strong correlation between navigation and CT measurement for both leg length (R=0.92) and offset (R=0.97) [6,24]. A major limitation of Renkawitz et al.’s paper is the use of cadaveric models. Renkawitz et al. later went on to compare measurements made using navigation in vivo and pre- and post-operative plain film radiographs. They found a mean difference of 0.40mm (±3.6) for leg length measurements and -1.0mm (±3.9) for offset measurements [25]. A further limitation is the use of non-absolute differences, whereby the mean of positive and negative difference values may negate each other and lower the mean difference. It was proposed that evaluating the mean absolute difference between the two measurements would be more representative. A more recent study by Clave instead used SterEOS (EOS Imaging, Paris, France) for comparative measurements to three navigation systems including BrainLab and demonstrated all three systems to be accurate to within 1mm [26].

It was deemed of interest that a clinical study assessing the accuracy of the BrainLAB Hip 5.1 navigation system be undertaken. This study assessed the accuracy of an imageless navigation system with a pinless femoral array (Hip 5.1, BrainLAB, Feldkirchen, Germany), compared to plain film radiographs, in measuring leg length and femoral offset changes in vivo. We hypothesized that the study would further support the findings of Renkawitz et al. [6, 24,25] that such measurements would be accurate.

This article was previously been presented as a meeting abstract at the International Society for Technology in Arthroplasty 2012 Annual Congress on October 5, 2012 [27].

## Materials and methods

This project has been granted ethics approval by the Human Research Ethics Committee (HREC) of Melbourne Health, Australia, project number QA2010112. The study group consisted of a prospective, consecutive group of patients recruited over a 12-month period (August 2010 to July 2011 inclusive). To be eligible, patients had to be undergoing elective navigated primary THA performed at either the Royal Melbourne Hospital or the Melbourne Private Hospital by the lead surgeon (A.B.) and/or his fellow (R.J.). Study size was defined by the planned 12-month recruitment period. Baseline demographic data were documented, including patient age, sex, body mass index (BMI), and pre-operative diagnosis.

**Figure 1:**
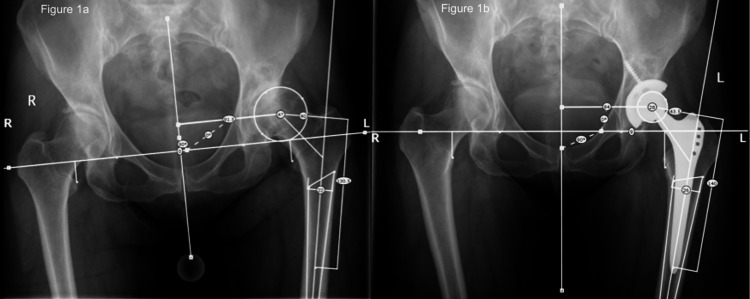
Pelvic radiographs depicting pre-operative (1a) and post-operative (1b) measurement of leg length and offset

Pre-operative planning involved measurement of existing limb length discrepancy as well as operative hip offset using the same computer system (OrthoView, LLC, Hampshire, UK), as shown in Figure 1. The goal for the surgeon was to achieve no residual limb length discrepancy when the contralateral hip was already replaced or unaffected by arthrosis. If the other hip had pathological shortening, then discretion was used to aim for a slightly longer leg, accounting for the shortening on the other side. The offset goal was to restore the patient's native femoral offset on the operative leg.

**Figure 2:**
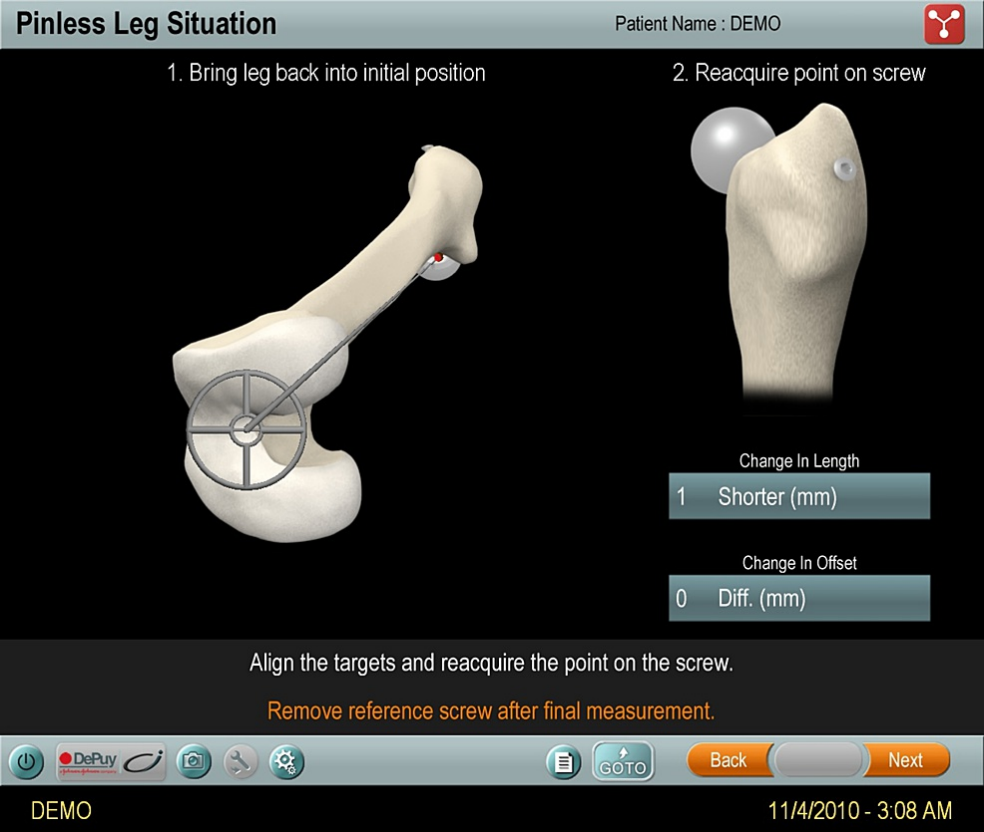
Intra-operative measurements made by the navigation system

A posterior approach in the lateral decubitus position was employed. Additional steps for the navigation system were required. Navigation was achieved using imageless navigation (Hip 5.1) with a pinless femoral array. This had been used by this surgeon since 2009. The iliac crest reference array was fixed using two 125x4mm Schanz screws. The navigation system intra-operatively recorded measurements of change made to leg length and offset after insertion of definitive implants (Figure 2).

All patients had pre- and post-operative digital radiographs with template markers, which were scaled and analyzed to provide radiographic measurements of the same changes. Leg length change was calculated as the change in distance from the inter-tear drop line to the tips of the lesser trochanter (Figure 1). Femoral offset was measured as the perpendicular distance from the center of the femoral head to the anatomical axis of the femur.

All data were entered into a spreadsheet, and using pre-operative and post-operative measurements of limb length and offset, as well as the change measured by navigation, the measured differences could be compared (Microsoft Excel 2008 for Mac, Microsoft Corporation, Redmond, WA, USA). Statistical analysis was carried out using STATA 10 (StataCorp LP, College Station, TX, USA).

## Results

A total of 37 patients were included: 34 with a primary diagnosis of osteoarthritis and three with avascular necrosis. The mean age of patients was 64 ± 14 years. There were 21 (56.8%) males. Mean BMI was 31.0 ± 5.1 kg/m^2^.

**Figure 3:**
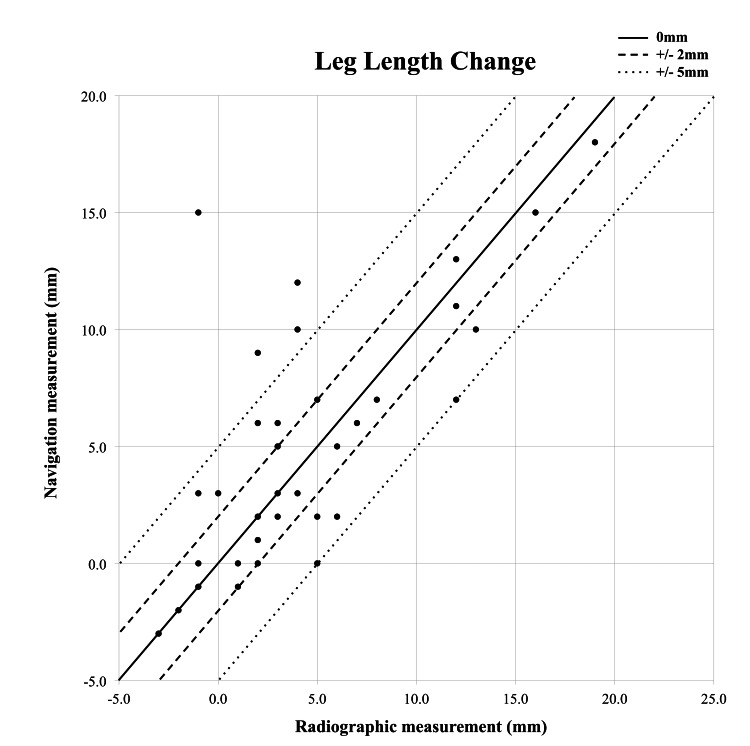
Scatter plot of radiographic and navigational measurements of leg length change

Leg length measurements made by the navigational system were demonstrated to have a strong correlation with radiographic measurements (R = 0.71; p < 0.0001) (Figure 3).

The mean difference between measurements was an overestimation by the navigation system of 0.73mm (Table 1). Table 2 and Figure 3 illustrate the accuracy of measurements.

**Table 1:** Comparison of radiographic and navigational measurements of leg length and offset changes

Parameter	Radiographic measurement, mean ± SD (mm; range)	Navigational measurement, mean ± SD (mm; range)	Mean absolute, difference ± SD (mm; range)
Leg length	4.4 ± 5.1 (-3.0 to 19.0)	5.1 ± 5.2 (-3.0 to 18.0)	2.6 ± 3.0 (0.0 to 16.0)
Offset	3.7 ± 6.9 (-13.5 to 22.0)	1.9 ± 5.3 (-9.0 to 13.0)	5.5 ± 4.7 (0.0 to 16.0)

**Table 2:** Accuracy of the navigation system in measuring leg length and offset changes to within 1, 2, and 5mm of radiographic measurements of change

Parameter	Within 1mm (%)	Within 2mm (%)	Within 5mm (%)
Leg length change	18 (49%)	24 (65%)	33 (89%)
Offset change	8 (22%)	13 (35%)	21 (57%)

**Figure 4:**
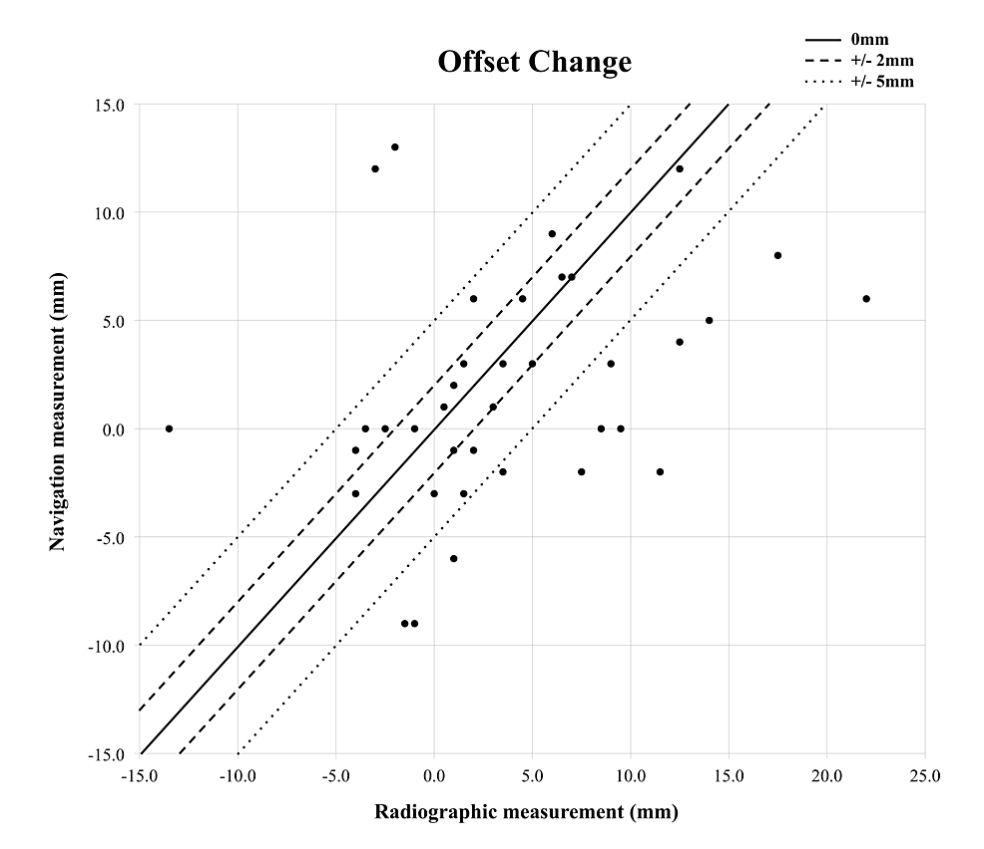
Scatter plot of radiographic and navigational measurements of offset change

Strength of correlation between the two methods was less for offset (R = 0.35; p = 0.035) (Figure 4).

On average, the navigational system under read offset by 5.5mm (Table 1). Examples of accuracy are demonstrated in Table 2.

## Discussion

It is critical to examine the ability of modern navigation software to provide surgeons with reliable intra-operative feedback on leg length and offset changes. This study is one of the few in vivo studies to assess the accuracy of an imageless navigation system with a pinless femoral array (Hip 5.1), compared to plain film radiographs. Leg length and offset restoration are important considerations in THA to minimize complications and improve outcomes. The navigation system employed in this study has been assessed in experimental studies by Renkawitz et al. in the past, using cadavers and saw bone models [6,24] as well as in vivo [25].

The study was limited in its sample size, and a greater sample population would increase the confidence in results. Due to the nature of the intervention, it was not possible to blind the surgeons to the navigation, and this is a limitation. The study was only conducted across two hospitals and two surgeons. There were also potential sources of error for measurements of leg length and offset. Firstly, the control data were obtained using plain radiographs. Using these for linear measurements is a potential source of error, caused by divergent X-ray beams, magnification errors, pelvic tilt or rotation, and femoral rotation [28,29]. When rotation of the femur occurs, leg length measurements are less influenced than offset, which can drastically be underestimated depending on limb rotation. As the control measurement was based on pre- and post-operative radiographs, the observed discrepancy between navigated and radiographic offset techniques may be from radiographic measurement inaccuracy rather than navigation error. CT imaging would be the gold standard for measurement, but in THA patients, this is not standard of care in the majority of hospitals, including the site of this study. Potential loosening and resultant movement of navigational arrays or reference screws is possible and may have inadvertently occurred, thereby introducing error to the navigation system readings [28].

For leg length, the navigation system showed a strong correlation with radiographic measures. In addition, the difference between the two methods of measurement showed a low mean. Most studies previously published examine the mean difference without using the absolute value, and thus when the over- and under-estimations are averaged, the difference appears falsely small. It was decided to examine the mean absolute difference to look at the average size of the navigation measurement discrepancy irrespective of direction from radiographic measurements. As a result, the mean difference of 2.6mm found in this study is slightly higher than differences described in the literature, which range from 0.2 to 1.3mm [6,22-25]. Furthermore, it was greater than the experimental mean difference of 0.5mm using the same navigation system in a cadaveric study and 0.5mm in vivo [6,24,25]. The difference here may be due to the use of more accurate reference measurements in the experimental studies where leg length and offset changes were measured from a fixed pelvis on a saw bone test bench or CT images. Once more, the in vivo study by Renkawitz et al. did not look at absolute values, and this may underestimate the true mean difference between navigation and radiographic measurements [6,24,25].

Accuracy within 5mm occurs in 89% of cases (Table 2), and this means that surgeons utilizing navigation can be reassured that the results are a close approximation of actual changes. This finding improves upon the observations of Kiefer and Othman, where a different navigation system was studied and found to be accurate within 5mm 83-85% of the time [23]. The correlation between methods was not as strong in this study (R=0.71) compared to the findings of Renkawitz et al. (R=0.92) [24] and Renkawitz et al. (R>0.80) [25]. A potential explanation for this is their use of CT imaging to measure the changes of leg length in the experimental study, which is more accurate than measurements from radiographs. There are also many other variables more easily controlled in their first study with a cadaver lab compared to live surgical patients, which may contribute to this difference.

For offset, the navigation system showed a correlation with radiographic measures, albeit not as strong as leg length. The mean difference of 5.5mm found in this study is larger than differences described in the literature, which range from 0.5 to 1.3mm [6,24,25,29]. Furthermore, it was once more a greater difference when compared with the experimental mean difference of -0.6mm using the same navigation system in a cadaveric study and -1.0mm in vivo [24,25].

The correlation between methods for measuring offset was not as strong in this study (R=0.35) compared to the findings of Renkawitz et al. (R=0.97) [24] and Renkawitz et al. (R>0.80) [25]. As discussed earlier, radiographic errors are more significant for offset measurement. In addition, the navigation workflow only allows for change in the overall distance from the greater trochanter to the midline, and thus medialization of the cup may be misrepresented as a reduction in offset by the navigation system but would not influence the radiographic measurements, potentially adding to discrepancies. Again, a potential explanation for the stronger correlation found in other studies would be their use of CT imaging to measure the changes of offset in the experimental study [6,24,25].

## Conclusions

Navigated computer-assisted technology for THA aims to enable orthopedic surgeons to successfully achieve desired leg length and offset and improve patient outcomes. This study demonstrates in vivo that an imageless, non-invasive navigation system is a useful tool for intra-operative leg length (accurate within 2mm) and offset measurement (accurate within 5mm) when compared to standard practice of plain film radiographs. Reporting of comparisons between navigated and radiographic measurements needs careful examination so that the mean of positive and negative differences does not falsely lower the average, and the authors recommend a novel approach used here for measuring the mean absolute difference. It is a reasonable intra-operative strategy that can be incorporated to improve leg length and femoral offset restoration in THA.
